# Mid-upper-arm circumference: a surrogate measure for BMI for age z-score to identify thinness among adolescent girls in Addis Ababa, Ethiopia

**DOI:** 10.3389/fnut.2024.1506576

**Published:** 2024-12-18

**Authors:** Solome Zebene, Gudina Egata, Demewoz Haile

**Affiliations:** ^1^Department of Nutrition and Dietetics, School of Public Health, College of Health Sciences, Addis Ababa University, Addis Ababa, Ethiopia; ^2^Institute for Health Metrics and Evaluation (IHME), Seattle, WA, United States

**Keywords:** mid-upper arm circumference, body mass index for age z-score, thinness, adolescent, Ethiopia

## Abstract

**Background:**

The mid-upper arm circumference (MUAC) is an anthropometric screening tool used to assess the nutritional status of individuals, offering a practical and feasible option in low-resource settings. However, the potential of MUAC as a screening tool for identifying thinness among adolescents remains underexplored.

**Objective:**

This study aimed to evaluate the accuracy of MUAC in identifying all forms of thinness among adolescent girls enrolled in selected schools in Addis Ababa, Ethiopia.

**Methods and materials:**

A representative sample of 913 female adolescent students was selected using a stratified sampling technique in a cross-sectional study. Socio-demographic data were collected via interviewer-administered questionnaires. Anthropometric measurements, including height, weight, and MUAC, were collected using a portable stadiometer, a battery-powered digital scale, and a non-stretchable MUAC tape, respectively. The Pearson correlation coefficient (r) assessed the relationship between MUAC, age, and body mass index for age (BAZ). The Receiver Operating Characteristic (ROC) curve was used to evaluate MUAC’s ability to classify those with or without thinness. The optimal threshold for adolescents aged 10–14 years was determined using Youden’s index.

**Results:**

A strong, significant positive correlation (r = 0.80) was observed between MUAC and BAZ. MUAC demonstrated good accuracy in detecting thinness and severe thinness (AUC = 0.86). Based on the highest Youden’s index values, MUAC cut-offs of 20.1 cm and 19.7 cm were identified to detect thinness and severe thinness, respectively. MUAC exhibited 92.1% sensitivity and 67.3% specificity in identifying moderate thinness and 95.9% sensitivity and 68.5% specificity for severe thinness among adolescent girls.

**Conclusion:**

MUAC showed good accuracy in predicting thinness and severe thinness, with comparable sensitivity but lower specificity than BAZ. Incorporating MUAC as a screening criterion for identifying thinness and severe thinness among female adolescents could be particularly beneficial in resource-limited settings such as Ethiopia.

## 1 Introduction

According to the World Health Organization (WHO), adolescence is defined as the age group between 10 and 19 years (1). This stage of life is characterized by increased demands for macronutrients and micronutrients to support accelerated physical and mental growth, development, and reproductive maturity. Poor nutritional status during adolescence can have long-lasting effects, exposing individuals and future generations to adverse health outcomes. Malnutrition often begins in the uterus and can persist across generations (2). As a result, malnourished female adolescents are more likely to become malnourished adults and give birth to low birth-weight infants. If those infants are girls, they are at greater risk of continuing the cycle by becoming stunted adults (3, 4).

For the assessment of the nutritional status of individuals, body mass index (BMI) is often used as a proxy measure to estimate body mass. However, it must also be taken into account that in practice, especially in many poor and middle-income countries, inadequate measuring devices such as a stadiometer make measuring height problematic, and skilled human resources are required to compute BMI in a population context (5). Hence, it would be important to consider the necessity for a surrogate marker that gives good results while also being easy to assess in a population setting.

The concept of using the arm circumference as the public health index for protein-calorie malnutrition (PCM) in the community was first employed in a nationwide survey in the Republic of Haiti in 1958, and since then, it has been applied in different study settings under different scenarios (6). Middle upper arm circumference (MUAC) is increasingly being used as an alternative tool to screen for undernutrition in adolescents and adults in low-resource settings, particularly among pregnant women and people living with HIV who are eligible for antiretroviral therapy (ART). This is because MUAC is easier and more convenient, requires less expertise, is simple and relatively inexpensive, and can be measured in both community and facility settings (7–9).

Recent studies have revealed that MUAC can be used to accurately identify moderate and severe forms of thinness in adolescents (10–14). More specifically, some studies revealed that the sensitivity and specificity tests proved the relationship between BMI and MUAC and that MUAC represents adolescent nutritional status with considerable efficiency and recommended further research to more establish that MUAC is a better and promising measure of adolescent nutrition, having the advantage of needing fewer resources for data collection (15, 16). Despite the evidence of a strong association, global MUAC cut-offs for adolescent thinness classification are yet to be established.

Different countries use different MAUC cut-offs for categorizing adolescent nutritional status for their respective programs due to differences in body size and body fat distribution across populations (17). Therefore, establishing standardized MUAC cut-offs for identifying cases of thinness among female adolescents would be of great help in reaching out to a larger community and strengthening and harmonizing programming in adolescent nutrition programs. However, little is known about the potential for MUAC to be utilized as a screening tool for determining thinness in adolescents. The present study evaluates the use of MUAC as an alternative tool for BMI z-score to detect all forms of thinness among adolescent girls.

## 2 Methods and materials

### 2.1 Study setting, design, and participants

A school-based cross-sectional study design was used among 913 randomly selected female adolescent students aged 10 to 14 years living in Addis Ababa, the capital city of the Federal Government of Ethiopia, which is located at 9°1′48″N latitude and 38°44′24″E and covers a total area of 540 km^2^. The city is subdivided into 11 sub-cities. The sub-cities are also divided into districts, and there are 126 districts in the city administration. According to the 2007 Census conducted by the Central Statistical Agency of Ethiopia (CSA), the city has a total population of 3,384,569, making nearly 4% of the country’s total, of whom 635,903 were adolescents, which is 23.2% of the total population (18). This study was conducted from April to June 2021 in selected governmental and non-governmental schools in Addis Ababa. Adolescents with visible physical deformities that can affect anthropometric measurements were excluded from the study.

### 2.2 Sample size determination

The diagnostic accuracy test sample size formula was used to calculate the required sample size (19) with the following assumptions.

The sample size required for sensitivity:

N1=zα22∗SN1−SN/L2∗P

The sample size required for specificity is as follows:

N2=zα22∗SP1−SP/L2∗1−P,

where n is the larger sample size between N1 and N2. Z α2 : Z value corresponding to a 95% level of significance = 1.96. P: Assumed prevalence. SN: Anticipated sensitivity. SP: Anticipated specificity. L: maximum clinically acceptable width or precision of the 95% confidence level.

Hence, assuming an anticipated sensitivity of 94.0% and specificity of 79.0% (12), using a prevalence of 21.3% (20), absolute precision of 5%, taking a design effect of 2, and a non-response rate of 10%, a maximum feasible sample size of 913 adolescents was used.

### 2.3 Sampling procedures

**Figure 1:**
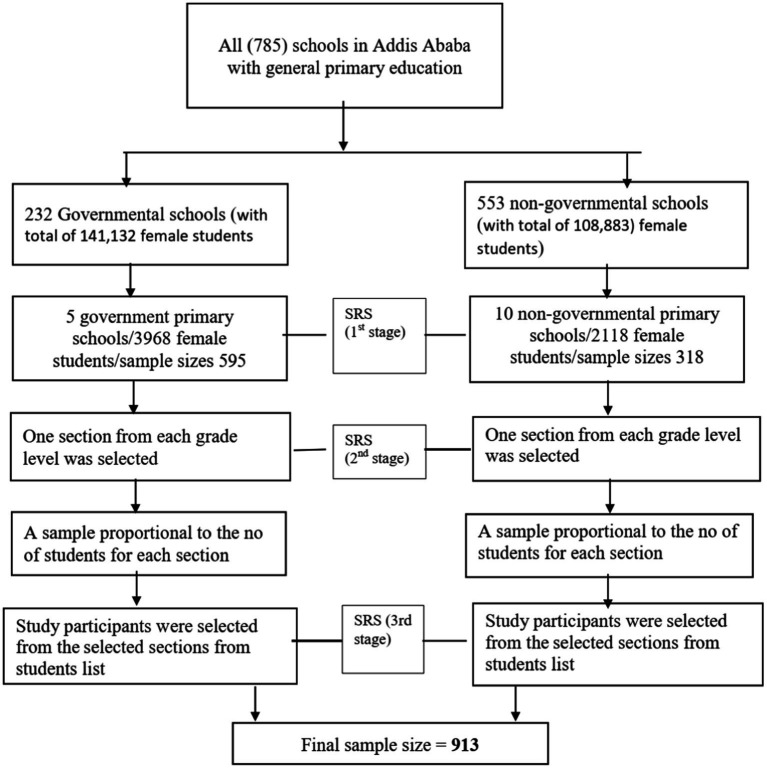
Sampling procedures of the study, 2022.

A stratified multistage sampling procedure was used to obtain a representative sample. The schools were first stratified by ownership into governmental (232 schools) and non-governmental (553 schools) categories. A simple random sampling was used to select 15 schools, of whom 10 belonged to private and 5 to governmental institutions, respectively. Then, the sample size was proportionally allocated to each selected school based on the number of students. One section from each grade level in selected schools was randomly chosen. Sampling frames (lists of students) were obtained from each school’s administrators. After proportionally allocating the number of study participants to each selected section, participants were randomly selected from these lists. Room teachers (i.e., those responsible for supervision of students) assisted in identifying the study participants (Figure 1).

### 2.4 Data collection

#### 2.4.1 Information on socio-economic, demographic, and environmental conditions

The data collection tool included socio-economic, demographic, environmental conditions, and anthropometry. Data were collected by eight trained data collectors and supervised by two supervisors who had previous exposure to research data collection and supervision, respectively.

Data on socioeconomic, demographic, and environmental conditions were collected using pretested, pretested, and structured questionnaires administered by the interviewer. All data were collected electronically using Kobo Collect software (an open-source Android app for collecting survey data), which is available at https://www.kobotoolbox.org/.

#### 2.4.2 Anthropometric measurements

All anthropometric measurements were taken twice, and the average was used for the analysis. Height measurement was carried out using a portable stadiometer with a sliding headpiece. Participants stood straight on a leveled surface without shoes, with heels together, arms hanging freely by the sides, palms facing forward, and fingers pointing downwards. The head, shoulder blades, buttocks, and heels touched the board’s wall. The measurement was taken from the sole of the feet to the vertex of the head and recorded to the nearest 0.1 cm. Weight was measured using a portable battery-powered digital scale without shoes and with minimum clothes (inner wear that does not affect the precision of measurements and can be adjusted for after measurement) and recorded to the nearest 0.1 kg. The MUAC was measured by a non-stretchable measuring tape on the non-dominant bare arm. This was determined by marking the mid-point between the tip of the lateral border of acromion and olecranon, then applying the tape measure to its middle point with the non-dominant arm hanging and relaxed. The tape was placed firmly but gently on the arm to avoid compression of soft tissue, and MUAC was recorded to the nearest 0·1 cm (21).

#### 2.4.3 Age determination

Although the grades of the students were used as proxies for age estimation, age was estimated by asking participants for their date of birth, followed by the month and year. The exact age was calculated as the difference between the interview date and the date of birth.

In this study, adolescents refer to individuals aged 10–14 years, which is consistent with the WHO definition of early adolescence (22). The 2007 WHO growth reference was used to classify adolescents with or without thinness/severe thinness using WHO Anthro Plus version 1.0.4 software. Adolescents with BAZ values corresponding to < −3 z-scores and < −2 to ≥ −3 z-scores were classified as severely thin and thin, respectively. Those with BAZ corresponding to ≥ 2 to < +1 z-scores and ≥ +2 z-scores were classified as overweight and obese, respectively (23).

### 2.5 Data quality assurance

The questionnaire was prepared in English, translated into the local language, and then translated back into English by another expert to ensure consistency and contextualization. Three days of training were provided to data collectors and supervisors about the study’s objective, measurement procedures, data collection, interviewing, and ethical issues. Practical exercises were performed on data collection techniques and procedures, as well as height, weight, and MUAC measurements, to ensure the quality of the field operation. The questionnaire was pretested on 46 school-going adolescents from non-selected schools in the city before the actual data collection. Amendments were made to the questionnaires as needed. Supervisors and investigators checked the collected data daily for completeness and consistency, providing feedback every morning before the next day’s activities. Measurement scales were carefully handled and calibrated every day before data collection. During weight measurement, the scales were carefully handled and periodically calibrated by placing the standard calibration weight of a 5 kg iron bar on the scale before use to ascertain accuracy. The data collector checked whether the scale reads 0.00 before weighing each participant. To improve the quality of the anthropometric data, an anthropometric standardization exercise was conducted. Based on the standardization exercise, technical error of measurement (TEM) was calculated among 10 adolescent girls. The intra-observer TEM for height ranged from 14.8–28.1%, for weight from 11.6–23.0%, and for MUAC from 8.9 to 26.07%. The inter-observer TEM was 12.5% for height, 23.6% for weight, and 35.7% for MUAC. In all cases, the computed TEM was within the acceptable range (24).

### 2.6 Statistical analysis

All statistical analysis was performed using STATA version 16.0 (25). Pearson correlation coefficient (r) was used to assess the relationship of MUAC with BAZ and age. It was interpreted according to Cohen’s criteria for r = 0.1–0.3 as weak, r = 0.3–0.5 as medium, and r = 0.5–1.0 as strong correlation (26). A paired t-test was used to analyze test–retest reliability for the 1st and 2nd mid-upper arm circumference measurements. For the variable MUAC, a Receiver Operating Characteristic (ROC) curve was plotted with the aim of obtaining a global measure of the accuracy of the test for the combination of all the possible cut-off points. The area under the curve (AUC) and its 95% confidence interval (CI) were calculated. An AUC of 1 indicates the ability to perfectly distinguish between those participants with and without thinness/severe thinness (perfect test), whereas an AUC of 0.5 suggests that these results were obtained just by chance (worthless test). The categories used to summarize the accuracy of AUC in ROC analysis were as follows: excellent (0.9–1), good (0.8–0.9), fair (0.7–0.8), poor (0.6–0.7), and fail (0.5–0.6) (27). Based on the curve coordinates, a cut-off point was selected for the variable MUAC. The optimal threshold for all age groups was determined from Youden’s index, which is the maximum value of J: j = sensitivity + specificity − 1. For a perfect diagnostic test, J = 1, whereas for a poor diagnostic test, J equals 0. Sensitivity, specificity, likelihood ratios (positive and negative), and positive and negative predictive values of MUAC were calculated for the proposed cutoff points for all age groups (28).

Positive likelihood ratios were calculated by dividing sensitivity by 1 − specificity, indicating the increased odds of a positive test result. Negative likelihood ratios were calculated by dividing 1 − sensitivity by specificity, indicating the odds of a case when a test is negative.

## 3 Results

### 3.1 Socio-demographic characteristics of study participants

Out of the sampled 913 adolescent students, a total of 884 participated in this study, making the overall response rate 96.8%. The majority (66.6%) of the respondents were from governmental schools, while the remaining 301 (33.4%) were from non-governmental schools. The majority of the study participants (58.8%) were in the age range of 10–12 years. The participants’ mean (±SD) age was 12.07 (±1.433) years. Over three-quarters (77.6%) of respondents were Orthodox Christian, followed by Muslim (10.9%), Protestant (9.8%), and Catholic (1.7%). Regarding the family size, the majority (61.43%) had a family size of five or more. Most of the respondents (40.9%) are self-employed fathers, while the proportion (30.6%) is less self-employed mothers. Similarly, 69.1% of the respondents belong to fathers and 50.4% to mothers whose educational status was secondary school and above, respectively (Table 1).

**Table 1:** Socio-demographic characteristics of the study participants Addis Ababa, Ethiopia, 2022 (n = 884).

Variables	Frequency	Percent
Age in years		
10–12	520	58.8
13–14	364	41.21
Mean age(±SD) years	12.07 (±1.433)	
Religion		
Orthodox Christian	686	77.6
Catholic	15	1.7
Protestant	87	9.8
Muslim	96	10.9
Family size		
≥5	543	61.43
<5	341	38.57
Paternal educational status		
Have no formal education	110	12.4
Primary school	163	18.4
Secondary school (grades 9–12 and above)	611	69.1
Paternal occupation		
Government/private employee	313	35.4
Self-employed	331	37.4
Daily labourer	59	6.7
Unemployed	46	5.2
Others	135	15.3
Mother’s educational status		
Have no formal education	202	22.9
Primary school (grades 1–8)	236	26.7
Secondary school (grade 9–12 and above)	446	50.4
Mother’s occupation		
Government/private employee	225	25.68
Self-employed	268	30.59
Daily labourer	48	5.48
Unemployed	316	36.07
Others	27	3.05
School enrolment		
Governmental	598	66.52
Non-governmental	301	33.48

### 3.2 Nutritional status of adolescents

**Figure 2:**
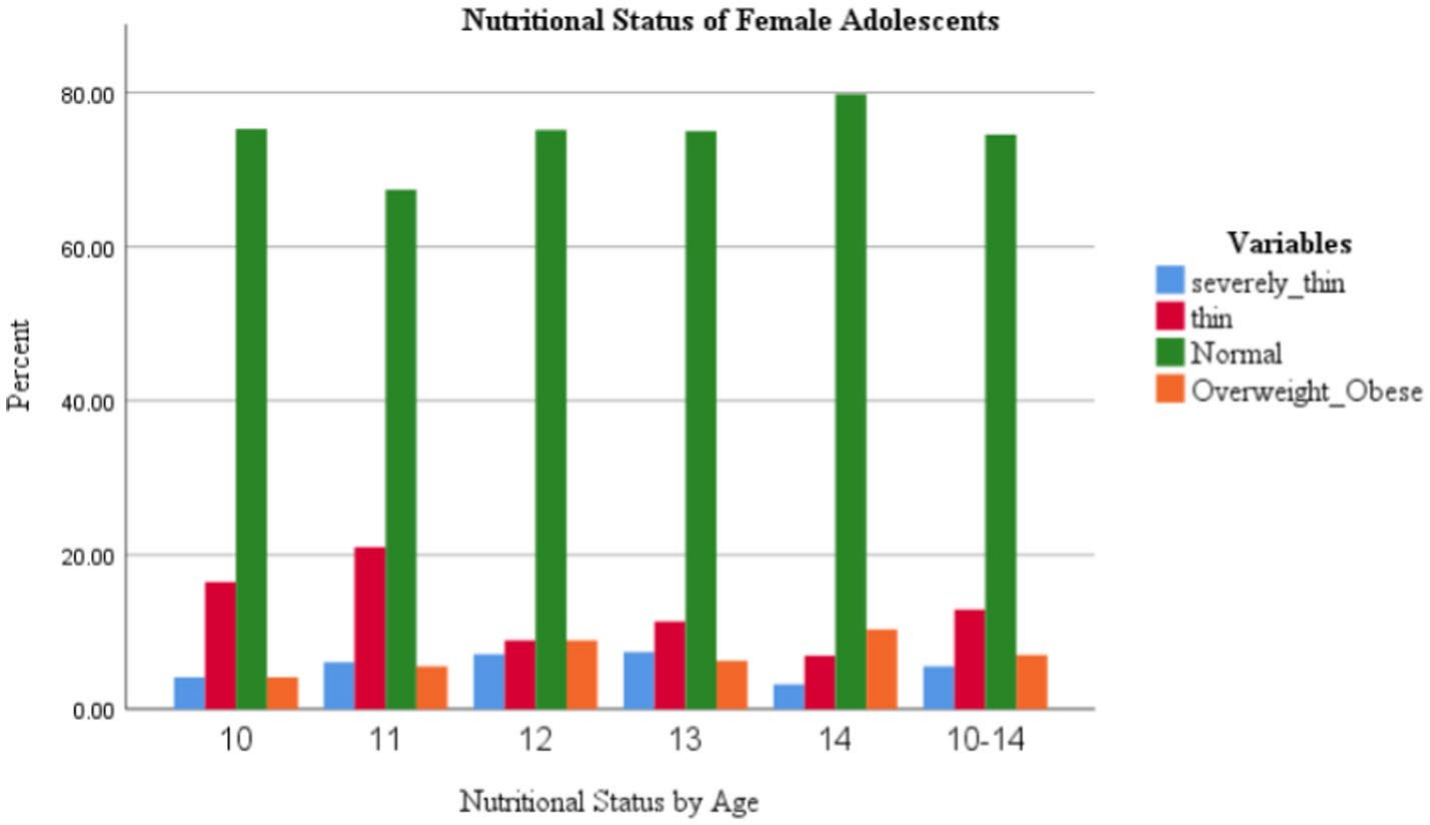
Age-wise distribution of adolescent school girls by their BAZ categories in Addis Ababa, Ethiopia, 2022.

The overall mean (± SD) weight, height, and MUAC among respondents in this study were 38.5 (±9.3) kg, 150 (±0.1) cm, and 20.7 (± 2.5) cm, respectively. On the other hand, the mean (± SD) BAZ was −0.84 (±1.30) (Table 2). The body mass index for age (BAZ) measurement of the study participants revealed that a quarter of the study participants had normal body weight. The overall magnitude of thinness was 18.0%. The magnitude of thinness, including severe thinness, was relatively higher (27.0%) among girls aged 11 years compared with other age groups in the early adolescent period. The magnitude of overweight and obesity (combined) was 7.0% in the total sample and relatively higher among adolescents aged 14 years (Figure 2).

**Table 2:** Mean of weight, height, mid-upper arm circumference (MUAC), and BMI for age (BAZ) of female adolescents Addis Ababa, 2022.

Age(years)	Weight (kg)	Height (cm)	MUAC (cm)	BAZ
	Mean (± SD)	Mean (± SD)	Mean (± SD)	Mean (± SD)
10	31.7 (−6.7)	142 (−0.1)	19.1 (−2.2)	−0.85 (−1.29)
11	34 (−7.0)	147 (−0.1)	19.8 (−2.1)	−1.18 (−1.26)
12	38.7 (−7.7)	151 (−0.1)	20.4 (−2.2)	−0.83 (−1.29)
13	41.8 (−9.5)	154 (−0.1)	21.3 (−2.0)	−0.93 (−1.36)
14	46 (−7.3)	155 (−0.1)	22.7 (−2.0)	−0.45 (−1.20)
Oct-14	38.5 (−9.3)	150 (−0.1)	20.7 (−2.5)	−0.84 (−1.30)

### 3.3 Test–retest reliability of MUAC

A perfect agreement was reached between the first and second MUAC measurements in all age groups (Table 3).

**Table 3:** Analysis of the reliability of MUAC among primary school adolescent girls, Addis Ababa, Ethiopia, 2022.

Age in years	MUAC 1st Mean (± SD)	MUAC 2nd Mean (±SD)	*p*-values	*t*-values
10	19.1 (2.2)	19.1 (2.2)	0.9975	−2.8439
11	19.7 (2.1)	19.8 (2.1)	0.9717	−1.9181
12	20.4 (2.2)	20.4 (2.3)	0.6660	−0.4297
13	21.2 (2.0)	21.3 (2.0)	0.9942	−2.5512
14	22.6 (2.0)	22.7 (2.0)	0.8179	−0.9097
Total	20.7 (2.4)	20.7 (2.5)	0.9972	−2.7750

MUAC, Mid-Upper Arm Circumference.

### 3.4 Relationship between MUAC and other anthropometric characteristics

The correlation between MUAC with BAZ and age revealed that MUAC had a strong, significant positive correlation with BAZ in all the subjects studied. Meanwhile, MUAC and age were moderately correlated (Table 4). Moreover, the results of the MUAC correlation coefficient with BAZ by the age of the study participants revealed that MUAC showed a strong correlation with BMI z-score across all age groups (Table 5).

**Table 4:** Results of overall Pearson correlation coefficient between MUAC with BMI for age *z*-score and age in years among adolescent girls in Addis Ababa, Ethiopia, 2022.

Characteristic	Mid-upper arm circumference	
	*r*	95% CI
Age in years	0.50	0.447–0.546
BMI *z*-score	0.80	0.775–0.822

**Table 5:** Results of Pearson correlation coefficient between MUAC and BMI for age *z*-score by different age groups among adolescent girls in Addis Ababa, Ethiopia, 2022.

Mid-upper arm circumference					
Age	10 years	11 years	12 years	13 years	14 years
Index	r(95% CI)	r(95% CI)	r(95% CI)	r(95% CI)	r(95% CI)
BMI *z*-score	0.88 (0.84–0.91)	0.89 (0.85–0.91)	0.81 (0.75–0.86)	0.92 (0.89–0.94)	0.82 (0.76–0.86)

### 3.5 Performance of MUAC to detect thinness, including severe thinness among female adolescents (BAZ of < −2)

**Figure 3:**
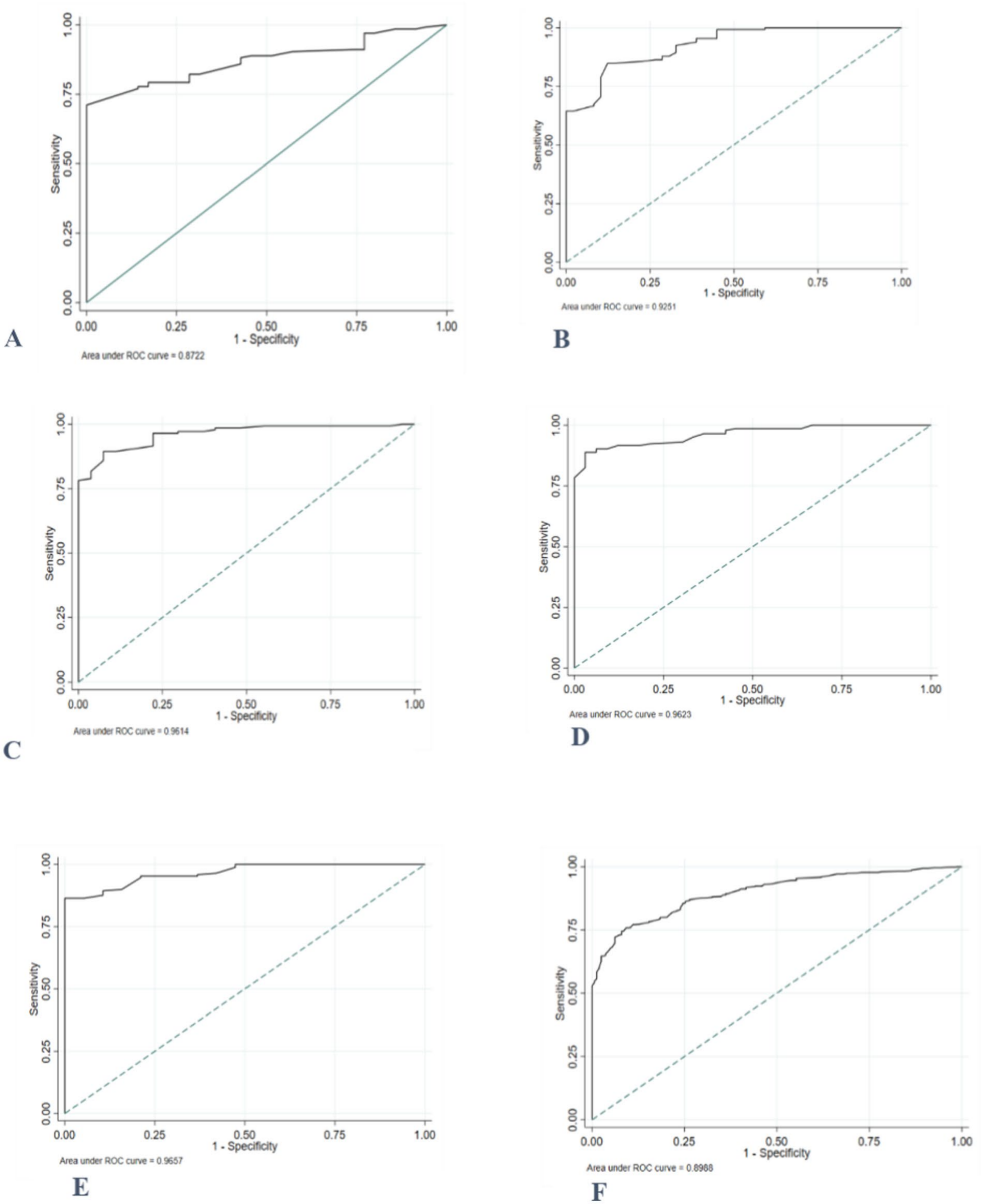
ROC curve depicting the ability of MUAC to classify thinness, including severe thinness among adolescent girls **(A)** 10 years old; **(B)** 11 years old; **(C)** 12 years old; **(D)** 13 years old; **(E)** 14 years old, and **(F)** 10–14 years old.

The AUC for MUAC to correctly identify thinness/severe thinness in girls during early adolescence against the reference based on BAZ ranged between 0.87 and 0.97. The performance of MUAC in detecting thinness, including severe thinness, was excellent in all age categories and ‘good’ for girls aged 10 years (Figure 3).

The MUAC value of ≤19.8 cm was the best optimal cut-off point to distinguish those adolescents with and without thinness/severe thinness with a higher Youden’s index of 0.67. The diagnostic accuracy measures, sensitivity, specificity, and negative predictive value (PPN) at this cut-off point were higher, while positive likelihood ratio (LR+), negative likelihood ratio (LR-), and positive predictive value (PPV) values were lower (Table 6).

**Table 6:** Diagnostic test accuracy measures for varying cut-offs of mid-upper arm circumference (MUAC) at different ages for predicting thinness, including severe thinness among primary school adolescent girls, Addis Ababa, 2022.

Age in years	10 years	11 years	12 years	13 years	14 years	10–14 years
SN (%) (95% CI)	85.7 (69.7–95.2)	87.8 (75.2–95.4)	92.6 (75.7–99.1)	90.3 (84.2–99.9)	94.7 (74.0–99.9)	90.8 (85.3–94.8)
SP (%) (95% CI)	77.0 (69.0–83.8)	84.8 (77.6–90.5)	89.4 (83.2–94.0)	88.8 (82.5–93.5)	86.4 (80.3–91.2)	75.9 (72.6–78.9)
PLR (%) (95% CI)	3.73 (2.66–5.23)	5.79 (3.82–8.79)	8.77 (5.37–14.3)	8.67 (5.44–13.8)	6.96 (4.69–10.33)	3.76 (3.28–4.32)
NLR (%) (95% CI)	0.19 (0.08–0.42)	0.14 (0.07–0.31)	0.08 (0.02–0.31)	0.03 (0.00–0.24)	0.06 (0.01–0.41)	0.12 (0.07–0.20)
PPV (%) (95% CI)	49.2 (40.9–57.6)	68.3 (58.6–76.5)	62.8 (50.5–73.1)	66.7 (55.7–76.1)	43.9 (34.5–53.7)	46.0 (42.6–49.4)
NPV (%) (95% CI)	95.4 (90.2–97.9)	94.9 (89.8–97.5)	98.4 (94.4–99.6)	99.2 (94.8–99.9)	99.3 (95.6–99.9)	97.3 (95.7–98.3)
Correctly classified	87.1	82.6	84.8	89.1	85.1	78.5
YI	0.71	0.73	0.82	0.86	0.86	0.67
MUAC cut off point (cm)	≤17.8	≤18.8	≤18.8	≤20.0	≤21.1	≤19.8

SN, sensitivity; SP, specificity; PLR, Positive Likelihood Ratio, NLR, Negative Likelihood Ratio, YI, Youden’s index; PPV, positive predictive value; NPV, negative predictive value.

### 3.6 The ability of MUAC to detect thinness among female adolescents

**Figure 4:**
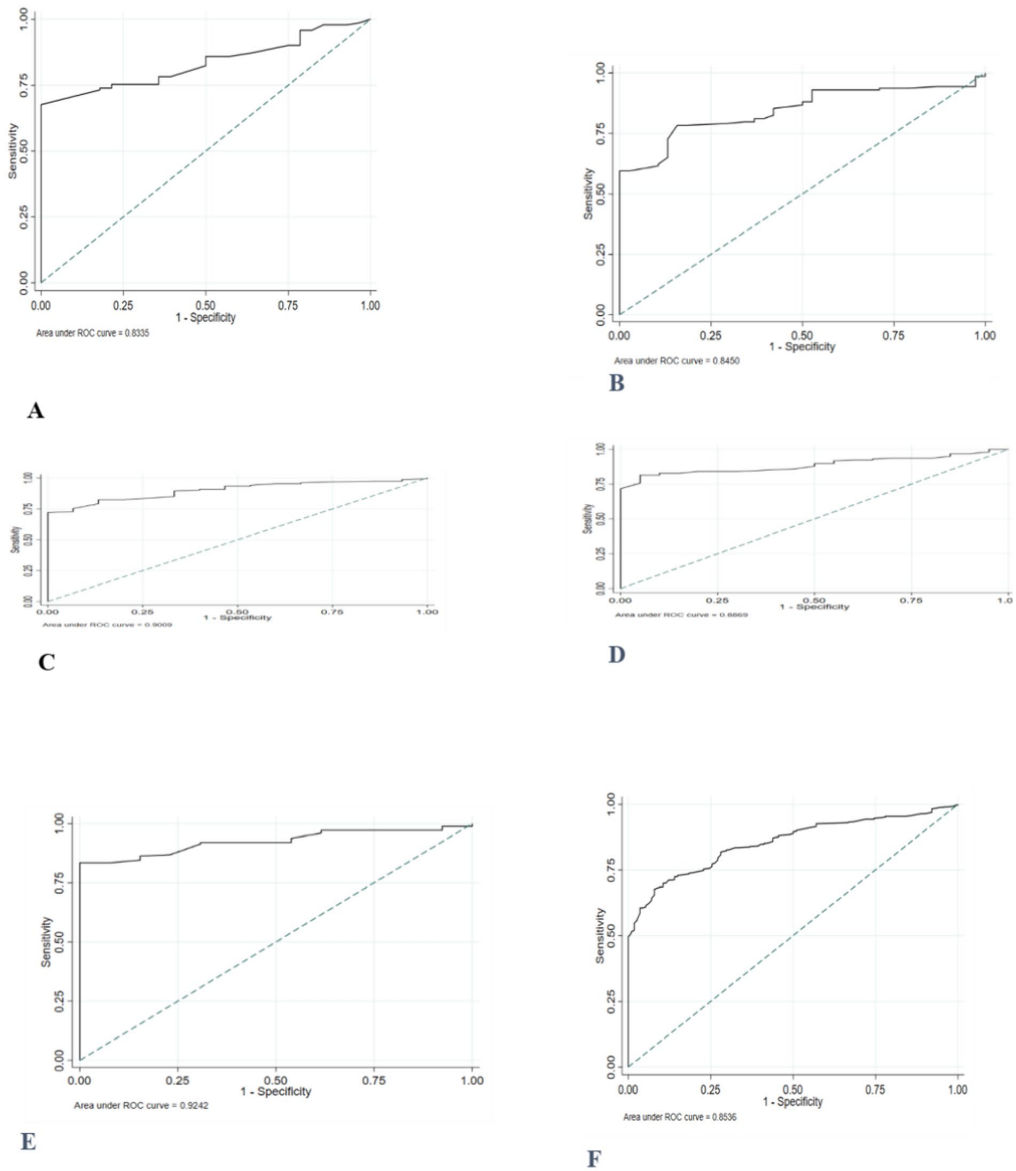
ROC curve depicting performance of mid-upper arm circumference for identifying thinness among adolescent girls **(A)** 10 years old; **(B)** 11 years old; **(C)** 12 years old; **(D)** 13 years old; **(E)** 14 years old, and **(F)** 10–14 years old.

The ROC curves comparing MUAC’s prediction of thinness in female adolescents throughout early adolescence to the gold standard/reference based on BMI z-score demonstrated that MUAC correctly detected thinness (AUC = 0.86). MUAC accurately predicted thinness in adolescents aged 12 to 14 years (Figure 4).

The proposed potential optimal MUAC cut-off to identify thinness was ≤20.1 cm. These cut-off points provide a sensitivity of 92.1, 95% CI: (85.5, 96.3%), and a specificity of 67.3, 95%CI: (63.7, 70.5%). Sensitivity and specificity were generally high for the optimal age-specific cut-points (73–100%). After stratification by age, higher optimal MUAC cutoffs were observed in the older age group (Table 7).

**Table 7:** Diagnostic test accuracy measures for varying cut-offs of mid-upper arm circumference (MUAC) at different ages for predicting thinness among primary school adolescent girls, Addis Ababa, 2022.

Age in years	10 years	11 years	12 years	13 years	14 years	10–14 years
SN (%) (95% CI)	82.1 (63.1–93.9)	84.2 (68.7–4.0)	100.0 (78.2–100)	95.0 (75.1–99.9)	84.6 (54.6–98.1)	92.1 (85.5–96.3)
SP (%) (95% CI)	73.2 (65.2–80.3)	78.3 (70.7–84.8)	72.1 (64.3–79.0)	81.4 (74.4–87.2)	86.3 (80.3–91.0)	67.3% (63.8–70.6)
PLR (%) (95%CI)	3.07 (2.22–4.24)	3.88 (2.76–5.46)	3.38 (2.78–4.62)	5.11 (3.62–7.20)	6.17 (3.98–9.56)	2.81 (2.51–3.16)
NLR (%) (95% CI)	0.24 (0.11–0.54)	0.20 (0.10–0.42)	0.00 (−)	0.06 (0.01–0.42)	0.18 (0.05–0.64)	0.12 (0.06–0.22)
PPV (%) (95% CI)	37.7 (30.5–45.5)	50.8 (42.3–59.2)	25.9 (21.3–31.0)	39.6 (31.7–48.0)	31.4 (22.8–41.5)	29.4% (27.1–31.9)
NPV (%) (95% CI)	95.4 (90.3–97.9)	94.9 (89.9–97.5)	100.0 (−)	99.2 (94.9–99.9)	98.7 (95.5–99.6)	98.3% (96.8–99.1)
Correctly classified (%)	74.7	79.6	74.6	65.1	84.1	70.8
YI	0.68	0.63	0.72	0.76	0.83	0.60
MUAC cut off point (cm)	≤17.8	≤18.9	≤19.6	≤20.0	≤21.1	≤20.1

SN, sensitivity; SP, specificity; PLR, Positive Likelihood Ratio; NLR, Negative Likelihood Ratio; YI, Youden’s index; PPV, positive predictive value; NPV, negative predictive value.

### 3.7 Diagnostic accuracy of MUAC cut-offs for thinness and severe thinness

**Figure 5:**
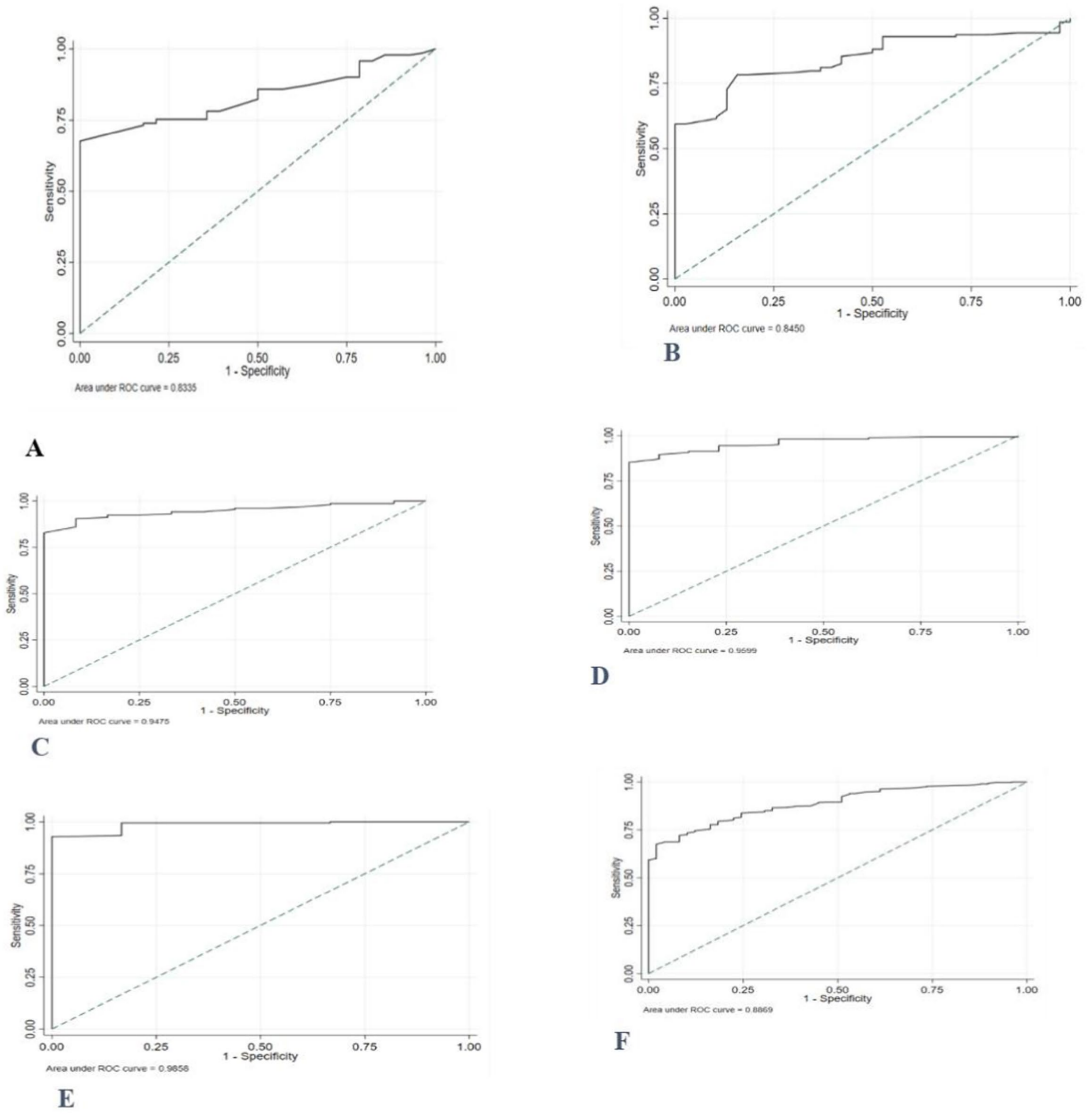
Receiver operating characteristics curve, depicting the performance of MUAC for identifying severe thinness among adolescent girls **(A)** 10 years old; **(B)** 11 years old; **(C)** 12 years old; **(D)** 13 years old; **(E)** 14 years old, and **(F)** 10–14 years old.

The accuracy of MUAC in diagnosing severe thinness among early female adolescents was good (0.87). Except for the age group of 10 years, the performance of MUAC in detecting severe thinness was excellent in adolescents of all ages (Figure 5).

The optimal MUAC cutoffs for the prediction of severe thinness were ≤ 19.7 cm. Notably, the MUAC cut-offs for severe thinness showed high sensitivity (95.9%), and the specificity was 68.5%. Furthermore, almost all sensitivities and specificities were high (>79.8%) after stratification for age. The percentage of individuals correctly classified using the proposed cut-off points ranged from 80.0 to 93.1% across age groups (Table 8).

**Table 8:** Diagnostic test accuracy measures for varying cut-offs of mid-upper arm circumference (MUAC) at different ages for predicting severe thinness among primary school adolescent girls, Addis Ababa, 2022.

Age in years	10 years	11 years	12 years	13 years	14 years	10–14 years
SN (%) (95% CI)	85.7 (42.1–99.6)	100.0 (71.5–100)	91.7 (61.5–99.8)	92.3 (64.0–99.8)	100.0 (54.1–100.0)	95.9 (86.0–99.5)
SP (%) (95% CI)	79.8 (72.8–85.6)	85.9 (79.7–90.7)	86.0 (79.6–91.0)	87.1 (81.0–91.8)	92.9 (88.1–96.1)	68.5 (65.2–71.6)
PLR (%) (95% CI)	4.23 (2.76–6.50)	7.08 (4.9–10.3)	6.54 (4.28–9.99)	7.16 (4.67–11.0)	14.00 (8.29–23.64)	3.05 (2.71–3.42)
NLR (%) (95% CI)	0.18 (0.03–1.10)	0.00 (0.00–0.00)	0.10 (0.01–0.63)	0.09 (0.01–0.58)	0.00 (−)	0.06 (0.02–0.23)
PPV (%), (95% CI)	15.4 (10.6–21.8)	31.4 (24.0–39.9)	33.3 (24.7–43.3)	36.4 (27.1–46.8)	31.6 (21.5–43.8)	15.2 (13.7–16.7)
NPV (%) (95% CI)	99.2 (95.5–99.9)	100.0 (−)	99.3 (95.4–99.9)	99.3 (95.6–99.9)	100.0 (−)	99.7 (98.7–99.9)
Correctly classified (%)	80.0	85.6	86.4	87.5	93.1	70.02
YI	0.75	0.86	0.83	0.54	0.93	0.66
MUAC cut off point (cm)	≤17.3	≤17.7	≤18.7	≤19.4	≤20.3	≤19.7

SN, sensitivity; SP, specificity; PLR, Positive Likelihood Ratio; NLR, Negative Likelihood Ratio; YI, Youden’s index; PPV, positive predictive value; NPV, negative predictive value.

### 3.8 The ability of MUAC to classify overweight, including obesity, among female adolescents aged 10–14 years

**Figure 6:**
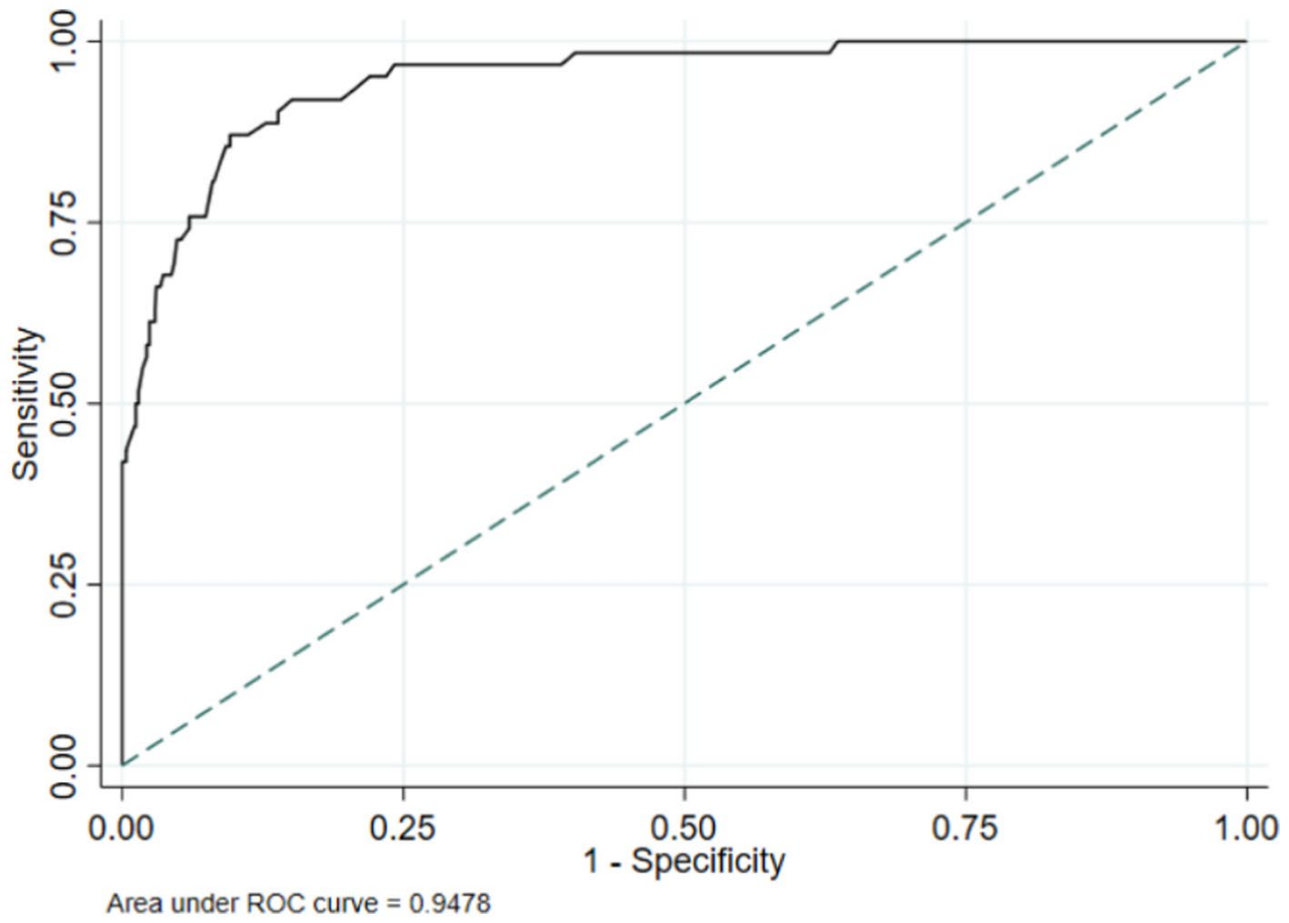
ROC curve depicting the ability of MUAC to classify overweight, including obesity, in 10- to 14-year-old female adolescents.

The ROC curve, which was generated based on BAZ > +1SD, revealed that the area under the receiver operating curve (AUC) of MUAC for adolescent girls (0.95) was high (Figure 6).

The MUAC value of 23.1 cm was found to be the suitable cut-off point for detecting overweight, including obesity, as it possessed the highest Youden index of 0.78. A sensitivity of 87.1% and specificity of 90.4% were found at this MUAC cut-off point (Table 8).

## 4 Discussion

Nutritional issues have been identified as significant public health concerns in Ethiopia. Many studies have been conducted to determine the extent and impact of malnutrition in various population groups (13, 29–33). Accordingly, adolescents are considered the vulnerable segments of the population who have not received adequate care to meet their nutritional demands. This study will be crucial in determining the nutritional status of “future mothers.”

The findings of this study indicated that the majority of subjects in the early adolescent (10–14 years) category have normal weight. The overall magnitude of thinness in this age category was 18.0%.

The magnitude of thinness observed in this study is consistent with a study conducted in the province of El Jadida in Morocco, which found that 18.8% of adolescents were underweight (34). This finding was lower than the results from studies conducted among adolescent girls in Adama City and Northern Ethiopia, which reported rates of 21.3 and 21.2%, respectively (20, 31). Nonetheless, the findings from the present study were higher than the report from a study in five rural districts of Amhara National Regional State, Ethiopia, which found that 13.6% of adolescent girls were underweight (33). Geographic, socio-economic, and behavioral variations of factors across these different settings may account for the difference.

The present study showed that MUAC had a significantly high positive correlation with BMI for age (MUAC versus BAZ: r = 0.80) but only moderately correlated with age (r = 0.5). This strong correlation is supported by findings from a study conducted on female adolescents in India, which found a positive correlation of 0.63 between BMI z-score and MUAC (11). Similar correlations of MUAC with BMI z-score were observed in studies conducted on adolescents in India and Ethiopia (r = 78, r = 0.81), respectively (12, 13). In contrast, a study from a rural area of Paschim Medinipur, India, reported a moderate correlation (r = 0.35) of MUAC with BMI among adolescent girls (14). Thus, MUAC may be a feasible nutritional screening tool in both clinical and field settings, especially where frontline professions (e.g., health extension workers) do not routinely use nutritional indicators like weight, height, and BMI z-score.

In this study, AUC values between 0.83 and 0.99 in various age groups showed that MUAC has accurate discriminatory performance among adolescents in identifying those with or without lower BMI-for-age z-scores. The results of this study are consistent with previous investigations. A study done in India reported an AUC ranging from 0.77–0.86 for adolescent girls aged between 10–14 years (MUAC vs. thinness/severe thinness) (11). Similarly, another study in the same country found AUC values ranging between 0·85 and 0·86, (*p* = 0·001) 0.93 and 0.98 for MUAC predicting thinness/severe thinness in a sample of Indian adolescent girls aged 10–19 years (12). These findings suggest that MUAC can serve as a complementary tool to BMI for age z-score in identifying thinness.

The present study proposed new MUAC cut-offs for diagnosing thinness, including severe thinness among school-going early adolescent girls. The optimal MUAC cut-offs to assess thinness and severe thinness in female adolescents were MUAC<20.1 cm and MUAC<19.7 cm, respectively. In comparison, a study from two eastern Indian states found MUAC cut-offs of 19.4 cm and 18.9 cm to be acceptable for identifying thinness and severe thinness among female adolescents (12). Another study among female adolescents in the rural and urban parts of India also showed that 19.4 cm and 18.3 cm were the best MUAC cut-off values to distinguish those with thinness and severe thinness, respectively (11). These variations in cut-off values might be attributed to differences in body composition among different ethnic groups (35).

Furthermore, when calculated for each age, cut-off values of MUAC to identify adolescents with or without thinness were between 17.5–20.9 cm and 17.0–20.0 cm for those with severe thinness in specific age groups. It was found that MUAC slightly increases as the age of adolescents increases. These findings suggested higher cutoff points than previous studies’ findings (11, 12). Because of significant physiologic age-related variations in body composition, the adoption of the same thinness/severe thinness cut-off point across different age groups and populations has been widely questioned (5). Therefore, the present study brought new evidence to shed some light on this controversy as it provides single age-specific MUAC cut-offs in addition to age-group-specific MUAC cut-offs to detect thinness and severe thinness among early female adolescents.

The performance analysis of the proposed cut-off points for MUAC carried out in this study revealed a good to excellent level of accuracy with sensitivity ranging from 92.1 to 95.9% and specificity from 67.3 to 68.5%. This specificity suggests that only a few adolescents at risk for thinness would be misclassified as normal. Given the greater health risks associated with thinness, it is more important to accurately classify those who are truly thin. Adolescents who are diagnosed as thin or severely thin using BMI z-score were also diagnosed as being thin or severely thin using MUAC. However, we feel that the optimal cutoff should not be established solely by an equation but rather with consideration of the context in which it will be applied. Selecting a cutoff requires a careful compromise between not missing any thin or severely thin adolescents (high sensitivity) and not misidentifying adolescents who do not need nutritional assistance (high specificity). In low-resource settings, using a cutoff with low specificity could further burden already overstretched health systems.

The accuracy of MUAC was also evaluated for all age groups. Sensitivity and specificity were in the range of 82.1 –100.0% and 72.1–92.9%, respectively, across stratified ages. These findings align with evidence from India, which showed high sensitivity and specificity for MUAC in predicting thinness/severe thinness in a sample of female adolescents aged 10–14 years (11, 12). In contrast, a study from Tanzania showed low sensitivity (40.0%) and high specificity (92.5%) for MUAC in predicting thinness/severe thinness in a sample of female adolescents aged 10–14 years (36). Similarly, a study among female adolescents in India further reported a 28.7% sensitivity and 96.46% specificity at the optimal MUAC cut-off point (10). This difference may be attributed to the varying reference values used in these studies.

This study has several important implications. First, it demonstrates that the anthropometric parameter MUAC is valid compared to the BMI z-score. Secondly, MUAC, as an alternative anthropometric measure, is a weightless tool that makes assessment more user-friendly, quick, and easy to use in the field. Additionally, this marker can be measured even in lying-down positions, making it suitable for individuals with disabilities. Therefore, this study demonstrates the potential of MUAC as a viable alternative anthropometric measurement in community-based screening and primary care settings.

The study also found that more than 78.5% of undernourished adolescents had thinness/severe thinness based on MUAC measurements. Since most Ethiopian adolescents have limited access to health facilities, relying solely on facility-based assessments may be insufficient to reach everyone. Screening in the community could be another way to reach out to the unreached group of the population.

The strengths of this study lie in its potential to provide a foundation for the utilization of MUAC as a screening tool for thinness and severe thinness among school-going female adolescents. Additionally, an anthropometric standardization exercise was conducted to enhance the quality of the anthropometric data. The TEM calculated from this exercise was within acceptable range in all cases (24). This reduces anthropometric measurement errors and ensures accurate and precise measurements.

Nevertheless, the findings of this study should be interpreted in light of its expected limitations. The use of MUAC in this particular study exclusively examined only on school-going female adolescents, which may not represent male adolescents. Therefore, the optimal MUAC cut-off values need to be cross validated with other community groups to establish its utility as a screening tool for thinness/severe thinness among all adolescents. Moreover, as the study was limited to one city, the findings may not be representative of the broader Ethiopian adolescent population.

## 5 Conclusion

The study authenticates the use of MUAC measurement, which is simple, inexpensive, easy to use, and non-invasive, as a prospective surrogate for BMI z-score to accurately identify thinness among female adolescents. The study also revealed that MUAC cut-off points were age-specific, with 19.7 cm for severe thinness and 20.1 cm for thinness as optimal values, independent of age. This anthropometric measurement showed good accuracy in predicting thinness and severe thinness with a comparable level of sensitivity and a lower specificity compared with the BMI z-score. Hence, the investigators believe that the introduction of MUAC as a criterion for easy and quick identification of thinness and severe thinness among female adolescents would be ideal in low-resource settings.

## Data Availability

The original contributions presented in the study are included in the article/supplementary material. Further inquiries can be directed to the corresponding author.
